# Nutritive Value and Degradation Kinetic Parameters of Three Plants for Feeding *Bradypus variegatus* Schinz, In Vitro Evaluation

**DOI:** 10.3390/ani14182645

**Published:** 2024-09-12

**Authors:** Igor Luiz Carvalho Máximo, Júlio Cézar dos Santos Nascimento, Gilcifran Prestes de Andrade, José Lypson Pinto Simões Izidro, Priscilla Virgínio de Albuquerque, Daniel Bezerra do Nascimento, Janerson José Coêlho, Marleyne José Afonso Accioly Lins Amorim, Alexandre Carneiro Leão de Mello, Romero Marcos Pedrosa Brandão Costa, Carlos Bôa-Viagem Rabello, Maria do Carmo Mohaupt Marques Ludke, Apolônio Gomes Ribeiro, Carolina Louise Nascimento de Santana, Ricardo Alexandre Silva Pessoa

**Affiliations:** 1 Departamento de Medicina Veterinária, Universidade Federal Rural de Pernambuco, Recife 52171-900, PE, Brazil; igor.luiz.7315@gmail.com; 2 Departamento de Zootecnia, Universidade Federal Rural de Pernambuco, Recife 52171-900, PE, Brazil; julio.nascimento@ufrpe.br (J.C.d.S.N.); danielbnascimento17@gmail.com (D.B.d.N.); alexandre.lmello@ufrpe.br (A.C.L.d.M.); maria.mmarques@ufrpe.br (M.d.C.M.M.L.); carolinalouisee@gmail.com (C.L.N.d.S.); ricardo.spessoa@ufrpe.br (R.A.S.P.); 3 Departamento de Morfologia e Fisiologia Animal, Universidade Federal Rural de Pernambuco, Recife 52171-900, PE, Brazil; gilcifran.andrade@ufrpe.br (G.P.d.A.); priscilla.albuquerque@ufrpe.br (P.V.d.A.); marleyneamorim@gmail.com (M.J.A.A.L.A.); 4 Departamento de Engenharia Agronômica e Ambiental, Universidade Regional do Cariri, Crato 63105-010, CE, Brazil; janerson.coelho@urca.br; 5 Instituto de Ciências Biológicas, Universidade de Pernambuco, Recife 50100-130, PE, Brazil; romero.brandao@upe.br; 6 Departamento de Ciência Animal, Universidade Federal da Paraíba, Areia 58397-000, PB, Brazil; apoloniogomes962@gmail.com

**Keywords:** Bradypodidae, chemical composition, *Inga* sp., in vitro kinetics, *Pterodon* sp.

## Abstract

**Simple Summary:**

Feeding sloths (*Bradypus* sp.) in captivity can be extremely challenging due to their individually selective folivorous diet and preferences for specific plant species. This selectivity complicates nutritional management and can lead to diseases associated with an unbalanced diet. By using an in vitro fermentation technique, we evaluated the nutritional characteristics of three feeds for captive sloths by incubating samples of these feeds with the stomach contents of sloths. Our findings indicate that the leaves of plants from the genera *Pterodon* sp. and *Inga* sp. show potential for feeding *Bradypus variegatus* sloths in captivity, with particular emphasis on the former, which demonstrated the best results for protein digestibility, fiber digestion rates, and total fermentation gas volume. We recommend careful attention to the fiber content of *Cecropia* sp. plants due to possible negative effects on dry matter intake. It is suggested that this plant be fed in combination with other feeds containing less fiber to allow for selection by the animals.

**Abstract:**

This study aimed to evaluate the nutritive value of three feeds (*Cecropia* sp., *Pterodon* sp., and *Inga* sp.) for sloths (*Bradypus variegatus*), based on nutritional composition and in vitro gas production. After a 14-day adaptation period to these feeds, approximately 500 g of gastric contents were collected from three female sloths, processed, and incubated with the food samples to evaluate digestibility and in vitro degradation kinetics. Regarding the nutritional composition, the neutral detergent fiber (NDFcp) content was higher with 404 g kg^−1^ DM (*p* = 0.001) in the leaves of *Cecropia* sp. The non-fibrous carbohydrate contents were greater with 499 g kg^−1^ DM in *Pterodon* sp. (*p* = 0.002). The greatest cellulose content (211 g kg^−1^ DM) was found in the leaves of *C. pachystachya*, as well as the lowest value of 143 g kg^−1^ DM for hemicellulose. Significant differences in the in vitro digestibility of crude protein (*p* = 0.041) were observed, with *Inga* sp. showing the highest value at 547 g kg^−1^ DM. In terms of kinetic parameters, *Pterodon* sp. exhibited higher total gas production (Vt) at 99 mL (*p* = 0.023) and digestion rates of fibrous carbohydrates (kdFC) at 0.0223%/h (*p* = 0.020) (*p* < 0.05). The leaves of *Pterodon* sp. and *Inga* sp. showed potential as suitable feeds for *B. variegatus*, while *Cecropia* sp. may have negative effects on dry matter intake due to its high NDF content, because of possible repletion effects on the stomach.

## 1. Introduction

The three-toed (*Bradypus* spp.) and two-toed (*Choloepus* spp.) sloths are obligate arboreal species in the Neotropics [1,2]. Both genera are primarily folivorous, but while the two-toed sloth is a generalist in its feeding habits, the three-toed sloth is more selective in its diet [3,4]. Studies in different habitats have shown that members of the Bradypodidae family prefer certain plant species, such as those from the Apocynaceae and Sapotaceae families [5], as well as Cecropiaceae, Clethraceae, and Clusiaceae [6].

Although some plant species are more consumed and common in the diet of the sloths than others, the factors that influence the choice of plant species remain unclear [7]. Differences in the physiological and nutritional needs of the diverse types of sloths can have a significant influence on these individual feeding choices [8]. In this context, the maintenance of sloths *Bradypus* spp. in ex situ environments is considered challenging, as their preferred types of feed are not always available, compromising the ideal nutritional status of these animals [9], leading to severe clinical disorders [10]. This restrictive feeding management leads to the development of weakness, dehydration, anorexia, weight loss, prostration, and immunosuppression [10], compromising the maintenance of these sloths in captivity.

Nevertheless, recent findings have demonstrated that free-living three-toed sloths are capable of consuming a great diversity and proportion of plant species [7], and also their diet can change or be adapted according to the tree species available in their habitat, suggesting that a greater variety of potential plant species can be consumed by these animals under captivity environments [11]. Digestibility assays using in vivo tests with *B. variegatus* corroborate this hypothesis, in which providing a greater diversity of plants in the diet of the sloths can favor their selective behavior, increasing nutrient intake and digestibility in mixed diets, up to 300 g kg^−1^ of neutral detergent fiber (NDF) in dry matter of the feed [12].

The nutritional assessment of plant species with potentialities for feeding sloths of the genus (*Bradypus* spp.) in captive conditions is essential to increase food options for these animals [13,14]. In different regions of Central and South America, the most common diets in ex situ conditions are based on leaves of *Cecropia* sp. [9], perhaps because they are known to have an ecological relationship or because they are one of the few plants with good acceptability by all *Bradypus*, regardless of individual preferences [3]. However, low levels of dry matter intake are observed when these plants are solely supplied [15], which may be associated with their high fiber content, especially lignin [13]. In contrast, leaves of *Inga* sp. and *Pterodon* sp. are among the species most consumed by three-toed sloths in the wild [7,11] and have a wide distribution in South America [16,17].

In nutritional terms, the leaves of *Inga* sp. were already reported to display values of 118 g kg^−1^ of crude protein (CP) and 335 g kg^−1^ of NDF [17], while *Pterodon* sp. values of 110 and 322 g kg^−1^ for the same parameters, respectively [16]. From the perspective of potential foods for feeding captive sloths, it is necessary to know their rates and extents of degradation using in vivo models that can show the potential of these foods in promoting an increase in the consumption and digestibility of DM and favoring the transit of digesta in sloths. These parameters are also linked to the soluble carbohydrate content of these foods, which have positively affected fermentation in the stomachs of sloths [12].

The objective of this study was to evaluate the nutritional composition, digestibility, and in vitro degradation kinetics of the leaves of *Cecropia* sp., *Inga* sp., and *Pterodon* sp. to feed sloths of the species *Bradypus variegatus* in captivity.

## 2. Materials and Methods

### 2.1. Diets, Animals, and Feed Analysis

**Figure 1:**
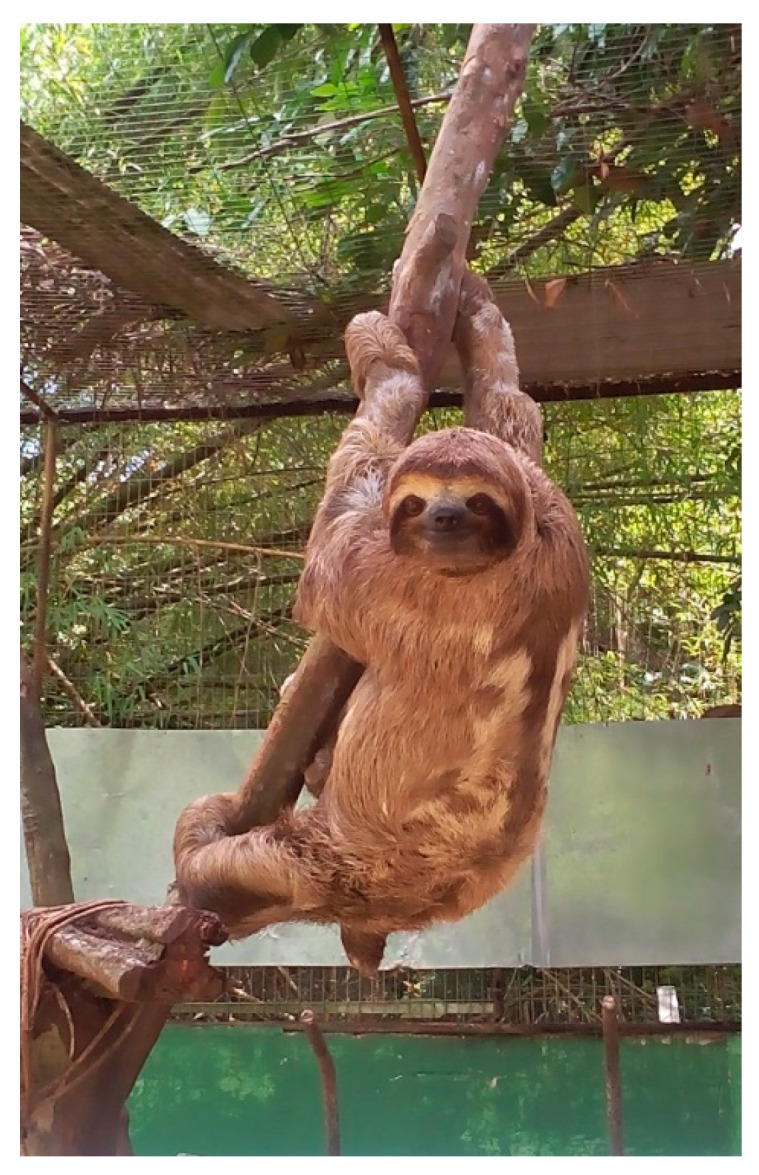
The species of *Bradypus variegatus* under captivity in the Dois Irmãos State Park, Recife.

This research was divided into two stages. The first stage began with food management of three sloths (2.8 ± 0.21 kg), females, of the species *B. variegatus*, kept in captivity in the Dois Irmãos State Park, Recife, Brazil (Figure 1). These three sloths were fed exclusively using the leaves of *Crecopia* sp. (Cecropiacea).

**Figure 2:**
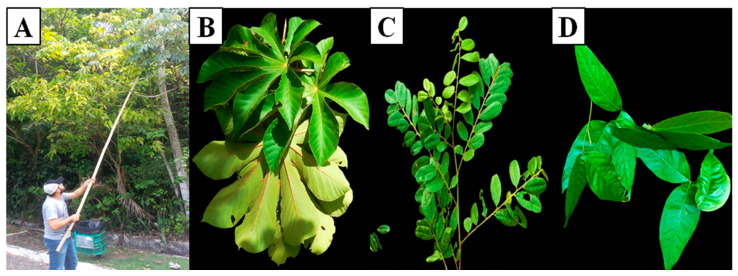
Collection method (**A**) to obtain leaves of *Cecropia* sp. Loefl (**B**), *Pterodon* sp. Vogel (**C**), and *Inga* sp. Mill (**D**), which were used in the diets provided to *B. variegatus*.

Over 14 days, the animals were adapted to a mixed diet solely composed of leaves of *Crecopia* sp., *Inga* sp. (Mimosoideae), and *Pterodon* sp. (Fabaceae) (Figure 2), in which 800 g of each feed was provided based on the fresh weight, considering up to 20% leftovers. There was no restriction or limitation of the dry matter intake by the animals. The plants were collected fresh, daily, close to the animals’ enclosure, and before each supply. Samples of these foods (leaves) were collected for bromatological composition and analysis of in vitro kinetic parameters.

The samples were pre-dried in a forced ventilation oven, at 55 °C, for 72 h and ground to 1 and 2 mm diameters, to estimate the contents of dry matter (DM), crude protein (CP), ashes, and ether extract (EE), according to Detmann et al. [18]. The fiber was quantified as neutral detergent fiber (NDFcp) and corrected for ashes and protein, according to Mertens [19]. The acid detergent fiber (ADF) was determined following Van Soest et al. [20] and lignin by the permanganate method proposed by Van Soest et al. [21]. Hemicellulose (HEM) was calculated as NDFcp—ADF; cellulose (CEL) as ADF—lignin.

### 2.2. Collection of the Digested Feed in the Stomach

After 14 days of feeding the diet, 500 g of gastric contents was collected from each animal, using an esophageal probe in the cranial portion of the stomach. The amount of gastric content collected for adult sloths is adequate, as it represented only 17% of body weight. An adult sloth weighing 3 kg can have up to 20–30% of its body weight as stomach digesta due to its slow metabolism, as reported by Foley et al. [13]. Additionally, during the experimental period, the animals consumed a mixed diet with an average intake of 384 ± 1.2 g/day. The samples were filtered through cotton fabric under CO_2_ injection and placed in a thermos bottle with hermetic closure, at 39 °C. They were then taken to the laboratory, filtered again under CO_2_ injection, and transferred to glass vials containing McDougall’s buffer solution. To avoid fermentation before inoculation, the flasks were kept in a refrigerator at 4 °C overnight. Five hours before inoculation, the flasks were removed from the refrigerator and taken to an oven at 39 °C until inoculation [22].

### 2.3. In Vitro Digestibility Test

The technique for evaluating the in vitro dry matter digestibility (IVDMD) was adapted from the method proposed by Tilley and Terry [23]. For the fermentative phase, 25 TNT (non-woven fabric) bags (5 × 12 cm) were incubated with 0.50 g of samples of each food using glass bottles (2 L capacity). These were heated to 39 °C in a McDougall buffer solution mixed with the filtered inoculum from the stomach of an adult sloth (400 mL). The medium was gasified using carbon dioxide (CO_2_), and the bottles were sealed with rubber stoppers and aluminum washers. The methodology proposed by Holden [24] was used, employing the artificial rumen equipment DAISYII Incubator (ANKOM^®^ Technology Corp., Fairport, NY, USA); the samples were kept in this equipment for 48 h. After this period, a solution containing 8 g of pepsin and 40 mL of 6N HCl was added to each flask, remaining for another 24 h under digestion. The bags containing the residues were washed with distilled water and dried in an oven at 105 °C until constant weight, and then weighed to estimate the IVDMD coefficient.

### 2.4. Digestion Kinetic Assay

The in vitro gas production test was carried out at the Embrapa Semi-arid Gas Production Laboratory, in Petrolina, Pernambuco. Approximately 1 g of each food sample was incubated anaerobically, at 39 °C, with 100 mL of stomach fluid of an adult sloth (buffered, buffer/fluid ratio 8:2), and sealed in glass vials (160 mL capacity) with rubber caps. Gas measurement was carried out following the semi-automatic gas production technique described by Mauricio et al. [25]. Pressure and volume readings were taken at increasing times of 0, 2, 4, 6, 8, 10, 12, 15, 24, 30, 36, and 48 h after incubation, using a pressure transducer connected to an outlet valve in the bottles.

The cumulative gas production data were analyzed using the bicompartmental model of Schofield et al. [26]: V(t) = Vf1/[1 + e^(2−4kd1(L-T)^] + Vf2/[1 + e^(2−4kd2(L-T)^], where V(t) represents the maximum total volume of gases produced; Vf1 the maximum volume of the gas for the faster digestible fraction; Vf2 the maximum volume of gas for the slow digestible fraction; kd1 the digestion rate for the fast digestible fraction; kd2 the digestion rate for the slowly digestible fraction; L the duration of the initial digestion events (latency phase), common to both phases; and T the fermentation time.

The bromatological composition and in vitro digestibility parameters were evaluated by analysis of variance, followed by the Tukey test, when the F test was significant, using a significance level of 5%. The parameters of the gas production models were estimated using nonlinear regression procedures, seeking the prediction equation that best fit the data obtained. Statistical analyses were performed using the software Statistical Analysis System (SAS) (Version 9.1, 2003).

## 3. Results

There was a significant difference (*p* < 0.05) in the average dry matter content of the leaves of *Inga* sp., displaying greater average values, followed by *Cecropia* sp. and *Pterodon* sp. (Table 1). Values for neutral detergent fiber were higher in *Cecropia* sp. (*p* < 0.05), followed by *Inga* sp. and *Pterodon* sp. (Table 1). Non-fibrous carbohydrate contents were greater in *Pterodon* sp. (*p* < 0.05), followed by *Inga* sp. and *Cecropia* sp. The cellulose content was higher in *Cecropia* sp. leaves compared to *Inga* sp. and *Petorodon* sp. (*p* < 0.05), while the latter had a higher hemicellulose content compared to *Cecropia* sp. (*p* < 0.05).

The in vitro digestibility results demonstrated a significant difference (*p* < 0.05) only for crude protein, with greater digestibility for *Inga* sp., followed by *Pterodon* sp. and *Cecropia* sp. (Table 2). However, there was no difference in the IVDMD and in vitro digestibility of the NDF between leaves (*p* > 0.05).

Regarding kinetic parameters, greater gas production was observed from the digestion of *Pterodon* sp. (*p* < 0.05), followed by *Inga* sp. and *Cecropia* sp., and these last two feeds did not differ from each other (Table 3). Regarding the volume of gases produced and the rate of digestion of fibrous carbohydrates, a statistical difference was observed between the feeds or leaves (*p* < 0.05). The volume of gas (vFC) was greater for *Pterodon* sp. (68.35 mL), followed by *Cecropia* sp. (61.69 mL) and *Inga* sp. (59.34 mL) (Table 3).

## 4. Discussion

The values of dry matter recorded in the present study corroborate the percentages observed in the literature for these leaves [12,15] and exceed the values indicated as minimum levels (250 to 350 g kg^−1^) for some ideal anaerobic fermentations [27]. No differences were observed for crude protein values between feeds and all presented a value above 7%, considered the minimum content to promote effective microbial fermentation in gastric fermenters [27].

The results of NDF for *Cecropia* sp. are in agreement with analytical data from other studies [12,13]. However, attention should be paid to the considerably high levels of NDFcp in this plant, as feeding sloths exclusively with *Cecropia* sp. may limit food intake. Studies have observed that values above 400 g kg^−1^ of NDF can limit food intake [15] and reduce the passage rate of the digesta in *Bradypus* sloths [13]. For foregut fermenters that consume fibrous diets, cellulose and hemicellulose are indispensable sources for obtaining liquid energy in the form of ATP, used for the maintenance and growth of the microbes in the digestive tract and the animal tissues [9].

Studies in animals with a similar gastric system indicate that the efficiency in the digestion and use of exogenous crude protein for the synthesis of microbial protein is related to the availability of energy in the fermentative environment, especially quickly and easily digestible non-fibrous carbohydrates or soluble carbohydrates, for example, simple sugars [28]. Therefore, it is reasonable to speculate that the greater digestibility of crude protein observed in this *Inga* sp. and *Pterodon* sp. may have occurred due to a greater supply of energy to the microbes involved in the digestion, and this energy may have originated from the greater content of NFC in these feed.

It was reasonably expected that, due to its higher levels of NDFcp, *Cecropia* sp. demonstrated lower total DM digestibility values; however, this premise was not confirmed. One of the factors that could justify such findings would be a greater solubility of cellulose and hemicellulose from *Cecropia* sp. leaves, which may have positively affected the total digestion of NDF in this feed, as observed in other herbivores that ferment vegetal organic matter in their foregut [29,30]. For foregut fermenters that consume fibrous diets, cellulose and hemicellulose are indispensable sources for obtaining liquid energy in the form of ATP, used for the maintenance and growth of the microbes in the digestive tract and the animal tissues [27].

The difference in total gas volume can be attributed to the fact that *Pterondon* sp. displayed a greater proportion of soluble carbohydrates compared to other food analyzed (Table 1). However, despite this difference, the average digestion rates estimated for non-fibrous carbohydrates (kdNFC) did not show a statistical difference among the feeds, as did the volume of gas from these carbohydrates (vNFC) (Table 3). The highest digestion rate of fibrous carbohydrates was also recorded for *Pterodon* sp. This was possibly related to the lower NDFcp content and higher non-fibrous carbohydrate values. As previously discussed, these components contribute significantly to the initial energy input in the fermentative medium [28].

Estimated digestion rates for fibrous carbohydrates in this study were slightly lower compared to assessments of fibrous feeds in other gastric fermenters, which ranged from 0.039 to 0.045% h^−1^ [31]. It is important to highlight that, unlike other herbivores, sloths from the genus *Bradypus* sp. have a stomach microbiota considered to be of low diversity, predominantly composed of the bacterial phyla Proteobacteria and Firmicutes [32]. This characteristic may, in part, have contributed to the relatively lower digestion rates observed. It is important to highlight that the leaves of the tested feeds presented relevant values of NDFcp and lignin, which is expected for leaves of tropical trees compared to most forage plants, and NDF consists of the carbohydrates cellulose and hemicellulose, in addition to the indigestible component lignin [27], so the association of these fibrous carbohydrates with lignin may have affected the rate of NDF digestion in these feeds.

On the other hand, the greatest contribution to the total volume of gas originated from the fermentation of fibrous carbohydrates in all feeds examined, according to the adjustment to the two-compartmental model (Table 3). In general, feeds with high NDFcp content and low NFC values are associated with lower digestion rates and lower gas production [33,34], which was not observed in this study. In all foods analyzed, the cumulative production of gas from non-fibrous carbohydrates was lower than the volume of gas from the fibrous fraction, indicating greater use of fibrous carbohydrates by microorganisms.

These events can be partially explained by the high content of cellulose and hemicellulose (NDF) in the feeds evaluated (Table 1). Such components, which ferment more slowly, probably contributed to the total gas production in longer periods. Normally, the fermentation of soluble carbohydrates occurs predominantly in the initial stages of fermentation to meet the energy needs of the microbiota, especially bacteria [28,35]. Furthermore, the notable levels of potentially digestible fiber in the foods evaluated may have favored the development of fibrolytic microbiota, intensifying the fermentation of fibrous carbohydrates.

## 5. Conclusions

Regarding the nutritive potentialities of the plant species tested to feed *B. variegatus*, the leaves of *Pterodon* sp. stood out, with greater values of crude protein digestibility, total volume of gas, and digestion rates of fibrous carbohydrates, which were influenced by its high values of non-fibrous carbohydrates and lower levels of NDF, demonstrating potential for feeding *B. variegatus* in captivity. The leaves of *Inga* sp. also presented potential for feeding *B. variegatus*, due to their adequate DM, CP, and non-fibrous carbohydrate values. However, caution is advised when feeding *Bradypus* sp. with *Cecropia* sp., due to the high NDF content of this plant, which might affect consumption, despite the absence of impacts on dry matter digestibility.

## Figures and Tables

**Table 1:** Nutritional composition of feed supplied to *Bradypus* sp.

Feed	DM	Ash	CP	NDFcp	NFC	LIG	CEL	HEM	EE
	(g kg^−1^)								
*Cecropia* sp.	385 b	97.6 a	95.4	404.2 a	367.26 c	19	211.2 a	143.2 b	37
*Pterodon* sp.	366 c	23 b	97.1	321 c	498.65 a	14	146.5 b	170.4 a	47
*Inga* sp.	401 a	17 b	86.8	343 b	486.43 b	16	138.6 b	167.2 a	43
*p*-value	0.002	0.001	0.223	0.001	0.002	0.178	0.001	0.002	0.157

DM (dry matter); CP (crude protein); NDFcp (neutral detergent fiber corrected for ash and proteins); NFC (non-fibrous carbohydrates); LIG (lignin); CEL (cellulose); HEM (hemicellulose); EE (ether extract). Means followed by different letters in the columns differ from each other using the Tukey test (*p* < 0.05).

**Table 2:** In vitro dry matter digestibility (IVDMD), crude protein (IVCPD), and neutral detergent fiber (IVNDFD) of leaves fed to *Bradypus* sp.

Feed	IVDMD	IVCPD	IVNDFD
	(g kg^−1^)		
*Cecropia* sp.	538.8	456.7 c	553.4
*Pterodon* sp.	552.4	522.7 b	574.5
*Inga* sp.	547.4	547.3 a	568.9
*p*-value	0.576	0.041	0.452

Means followed by different letters in the columns differ from each other using the Tukey test (*p* < 0.05).

**Table 3:** Digestion rates and volume of in vitro gas production from feed fractions.

Parameter	*Cecropia* sp.	*Pterodon* sp.	*Inga* sp.	*p*-Value
Vt (mL)	86.98 b	99.13 a	87.46 b	0.023
vNFC (mL)	25.29	30.78	28.12	0.251
kdNFC (%/h)	0.0364	0.0375	0.0278	0.376
L (h)	2.7	3.1	2.9	0.541
vFC (mL)	61.69 b	68.35 a	59.34 b	0.014
kdFC (%/h)	0.0166 b	0.0223 a	0.0189 b	0.020
Eq	y = −0.014x^2^ + 1.7003x + 8.9947	y = −0.0168x^2^ + 2.1851x + 2.9791	y = −0.017x^2^ + 1.8444x + 7.8076	-
R^2^	0.9777	0.9850	0.9730	-

Vt (total volume of gas); vNFC (volume of gas in the non-fibrous carbohydrates fraction); kdNFC (digestion rate of the non-fibrous fraction (%/h); L (lag time); vFC (volume of gases in the fibrous fraction); kdFC (digestion rate of the fibrous fraction); Eq (regression equation); R^2^ (coefficient of determination). Means followed by different letters in the lines differ from each other using the Tukey test (*p* < 0.05).

## Data Availability

The data that support the findings of this study are available on request from the corresponding author.
